# Impact of nutritional status on heart failure mortality: a retrospective cohort study

**DOI:** 10.1186/s12937-021-00753-x

**Published:** 2022-01-06

**Authors:** Nafiz Abdoul Carime, Jonathan Cottenet, Guillaume Clerfond, Romain Eschalier, Didier Quilliot, Jean-Christophe Eicher, Bertrand Joly, Catherine Quantin

**Affiliations:** 1 Biostatistics and Bioinformatics department (DIM), Dijon University Hospital, Dijon, France; University of Bourgogne Franche-Comté, Dijon, France; 2 Cardiology Department, CHU Clermont-Ferrand, Clermont-Ferrand, France and Université Clermont Auvergne, CHU Clermont-Ferrand, CNRS, SIGMA Clermont, Institut Pascal, F-63000 Clermont-Ferrand, France and F-CRIN, INI-CRCT, Nancy, France; 3 Department of Gastroenterology and Inserm NGERE U1256, University Hospital of Nancy, University of Lorraine, 1 Allée du Morvan, 54511 Vandoeuvre-lès-Nancy, France; Nutritional Assistance Department, University Hospital of Nancy, University of Lorraine, Vandoeuvre-lès-Nancy, France; 4 Cardiology Department. University Hospital of Dijon, Dijon, France; 5 CHPCB Paray-le-Monial General Hospital, Paray-le-Monial, France; 6 Inserm, CIC 1432, Dijon, France; Dijon University Hospital, Clinical Investigation Center, clinical epidemiology/ clinical trials unit, Dijon, France; 7 Biostatistics, Biomathematics, Pharmacoepidemiology and Infectious Diseases (B2PHI), INSERM, UVSQ, Institut Pasteur, Université Paris-Saclay, Paris, France

**Keywords:** Heart failure, Obesity, Malnutrition, Nutrition disorders, Medico-administrative database

## Abstract

**Background:**

Chronic heart failure (CHF) is one of the most common causes of mortality in industrialized countries despite regular therapeutic advances. Numerous factors influence mortality in CHF patients, including nutritional status. It is known that malnutrition is a risk factor for mortality, whereas obesity may play a protective role, a phenomenon dubbed the “obesity paradox”. However, the effect of the obesity-malnutrition association on mortality has not been previously studied for CHF. Our aim was to study the effect of nutritional status on overall mortality in CHF patients.

**Methods:**

This retrospective, multicenter study was based on a French nationwide database (PMSI). We included all CHF patients aged ≥18 years admitted to all public and private hospitals between 2012 and 2016 and performed a survival analysis over 1 to 4 years of follow-up.

**Results:**

Malnutrition led to a significant decrease in life expectancy in CHF patients when compared with normal nutritional status (aHR=1.16 [1.14-1.18] at one year and aHR=1.04 [1.004-1.08] at four years), obese, and obese-malnutrition groups. In contrast, obesity led to a significant increase in life expectancy compared with normal nutritional status (aHR=0.75 [0.73-0.78] at one year and aHR=0.85 [0.81-0.90] at four years), malnutrition, and obese-malnutrition groups. The mortality rate was similar in patients presenting both malnutrition and obesity and patients with normal nutritional status.

**Conclusions:**

Our results indicate that the protective effect on mortality observed in obese CHF patients seems to be linked to fat massincrease. Furthermore, malnourished obese and normal nutritional status patients had similar mortality rates. Further studies should be conducted to confirm our results and to explore the physiopathological mechanisms behind these effects.

**Supplementary Information:**

The online version contains supplementary material available at 10.1186/s12937-021-00753-x.

## Background

Chronic heart failure (CHF) is defined as a structural or functional abnormality of the heart leading to insufficient oxygen and nutrient intake to meet the metabolic needs of different organs [1]. Heart failure can be a complication of different cardiovascular diseases such as ischemic heart disease, cardiomyopathies, hypertension, or valve diseases. The worldwide prevalence of CHF is currently estimated at 8.52 per 1,000 inhabitants, and it is responsible for an estimated 11.61 per 1,000 years of healthy life lost due to disability (YLD) [2]. In France, its prevalence is currently estimated at 2.3%, and increases as the population ages [3]. Among patients managed in France for heart failure in 2014, 18% died in 2015, and the mortality caused by heart failure is estimated at 10%.

In addition, chronic heart failure progresses irregularly, with acute phases of decompensation that are a common cause of hospitalization, leading to more than 160 000 hospitalizations per year in France. The prognosis for decompensated heart failure remains poor [3]. Weight loss or cachexia are aggravating factors in patients who already have a bleak prognosis in terms of survival [4].

Obesity is also a major public health issue, with a prevalence that continues to rise at an alarming pace in industrialized countries. It has been established that obesity is a risk factor for the development of heart failure but that it becomes a protective factor for survival once heart failure is established [5]. This effect is known as the “obesity paradox”.

Malnutrition is defined as a metabolic state resulting from chronic inadequacy between anabolism and catabolism, where insufficient nutritional intake causes a chronic catabolic state resulting in weight loss [6]. This change in metabolism is accompanied by an increased level of IL-2 and IL-6, TNFalpha, increased sympathetic stimulation and cortisol/DHEA ratio, and stimulation of the renin-angiotensin-aldosterone system. All these factors contribute to anorexia, weight loss and increased muscle catabolism [7].

In industrialized societies, malnutrition is frequently observed in individuals with chronic diseases, where energetic spending is increased due to increased metabolic turnover and nutritional intake is reduced due to loss of appetite. Malnutrition-induced conditions such as sarcopenia, defined as muscle weakness associated with low muscle quantity and quality [8], are well-established factors for poor prognosis for chronic disease [9], where they constitute a therapeutic challenge. This is especially true in geriatric patients, in whom these conditions are most prevalent [10]. For CHF, it has been proven that sarcopenia and cachexia are associated with lower survival [9, 11].

Obesity and malnutrition are two conditions that can be associated in the same patient [12–14]. In the case of such an association, the weight loss induced by malnutrition predominantly affects muscle mass [15]. We can therefore identify a “malnutrition-obesity” group of heart failure patients who have still not been extensively studied, at least in terms of survival. In summary, the effect of nutritional status on CHF mortality is complex, with malnutrition constituting a risk factor, obesity possibly playing a protective role, and the combined effect being mostly unknown. This “obesity paradox” still has no consensual physiopathological explanation in the literature [16–18], which was the main motivation for this study. Our goal was thus to perform a comparative survival study of a population of CHF patients presenting obesity alone, malnutrition alone, obesity and malnutrition combined, or neither obesity nor malnutrition. We hypothesized that, in CHF patients, malnutrition would have a deleterious effect, obesity would have a protective effect, and the malnutrition-obesity association would have an intermediary effect.

## Methods

### Data source

The French hospital discharge abstract database (*Programme de Médicalisation des Systèmes d’Informations* (PMSI)) contains nationwide data on hospitalizations. Medical and administrative data are systematically collected at each hospital admission (public or private), and each patient is identified with a unique anonymous code. The PMSI data consists of primary and associated diagnoses during hospitalization encoded using WHO International Classification of Diseases and Related Health Problems, 10th revision (ICD-10), and procedures performed during all hospital stays using the French common classification system for medical procedures (*Classification commune des actes médicaux*). Hospital activity and funding is estimated from PMSI data, thus ensuring the exhaustivity of this database. These hospital data have been used in medical research for about 20 years, and their quality has been confirmed in recent studies [19–23]. This study was approved by the National Committee for Data Protection (declaration of conformity to the methodology of reference 05 obtained on 7/08/2018 under the number 2,204,633 v0) and was conducted in accordance with the Declaration of Helsinki.

### Population

This study was a retrospective multicenter study based on nationwide PMSI data. We included all patients aged ≥18 years admitted to all French acute care hospitals (public or private) over a period of 5 years (2012-2016), with a main diagnosis of heart failure (ICD-10 code I50). To include only incident cases, patients with an ICD-10 code I50 of heart failure (main or associated diagnosis) in the previous 4 years were excluded.

### Outcomes and follow-up

The main outcome was hospital mortality occurring 1 to 4 years after the first CHF diagnosis. Four-year follow-up on mortality was possible for patients included in 2012 and followed until 2016 (end of our study). The cohort followed-up for 3 years was constituted of patients included in 2013 (followed-up until 2016) or 2012 (followed-up until 2015). By the same logic, 2-year follow-up was possible for patients included in 2014, 2013 or 2012, and 1-year follow-up was possible for patients included in 2015, 2014, 2013 or 2012.

### Variables studied

#### Variables of interest

Our primary objective was to determine if nutritional status had an influence on the mortality of patients diagnosed with heart failure, and to quantify this influence after correcting for confounding factors. We thus divided our population into 4 groups with differing nutritional statuses: (1) a control group of patients who were neither obese nor malnourished, (2) an obesity group, (3) a malnutrition group, and (4) an obesity-malnutrition group.

Malnutrition was identified using ICD-10 codes from the hospital stay for CHF. The diagnosis of malnutrition was based on the presence of at least one of the following in patients under 70 years of age: weight loss ≥10% compared to a prior value (or 5% in 1 month); BMI ≤17 kg/m²; albumin < 30 g/L or prealbumin < 110 mg/l (if no inflammatory syndrome). For patients aged 70 years and older, one of the following criteria was required: weight loss ≥5% in 1 month, or ≥10% in 6 months; BMI < 21 kg/m2; albumin level < 35 g/l. We also classified malnutrition according to severity: severe malnutrition (E40 to E43), moderate malnutrition (E440), slight malnutrition (E441) and malnutrition not otherwise specified (E46). The use of these codes is based on different criteria and coding rules (Supplementary Text 1).

We used a similar procedure to identify obesity, using ICD-10 codes E66 and excluding overweight-specific codes (E6603, E6613, E6683, E6693). The diagnosis of obesity was based on a BMI ≥ 30 kg/m2. Obesity was also classified according to severity: massive obesity (BMI ≥ 50 kg/m2), morbid obesity (BMI 40 to 49 kg/m2), standard obesity (BMI 30 to 39 kg/m2), and obesity not otherwise specified. The classification of the ICD-10 codes used is given in Supplementary Table 1.

Both obesity and malnutrition were recovered at baseline.

#### Mortality

Mortality was established according to the variables available in the national medical-administrative database (PMSI). This information is contained in the variable “mode of discharge” (alive or deceased).

#### Confounding variables

The patient characteristics considered as confounding variables, including age, gender, the etiology of CHF (Ischemic, dilated or hypertensive cardiomyopathy), the presence of comorbidities such as diabetes, hypertension, dyslipidemia, kidney failure, chronic obstructive pulmonary disease (COPD), and the presence of infection and shock during the hospital stay, were added to our model in order to perform a statistical adjustment. These confounding variables were identified as mortality risk factors in a previous study [24]. A complete list of the confounding variables is provided in Supplementary Table 2.

All of these confounding variables were recovered at baseline.

### Statistical analysis

The different variables studied were compared using the Chi-2 test or Fisher’s exact test for categorical data and Student’s t-test or Mann-Whitney test for continuous data.

To study the influence of the variables of interest defined above (all dichotomized) on mortality on a follow up period ranging from 1 to 4 years, we first used a Kaplan-Meier method.

To take into account other factors, we then performed a survival analysis using a Cox proportional hazards regression model. The proportional hazard assumption was studied using the Kaplan-Meir curves, and interactions with time were taken into account when this assumption was violated. Hazard ratios (HRs) and 95% confidence intervals (CIs) were estimated after adjustment for potential confounders, using time from index hospitalization for CHF to in-hospital death. Outcome was measured over a 4-year period after index hospitalization for CHF. Individuals were censored at death, or the latest all-cause hospitalization for those who did not die. We followed individuals until in-hospital death, or the end of the 4-year follow-up period.

A sensitivity analysis was performed by limiting the follow-up to one year in order to have a larger number of incident cases and therefore a higher statistical power.

Another sensitivity analysis was conducted by excluding ICD-10 codes E660 (Obesity due to excess calories).

The statistical significance threshold was set at < 0.05. All analyses were performed using SAS (SAS Institute Inc, Version 9.4, Cary, NC).

## Results

Our study included a total of 619 805 patients diagnosed with CHF (Supplementary Fig. 1), whose characteristics are presented in Table 1. We observed an increase in malnutrition from 2012 to 2016 (from 8.4% to 2012 to 12.3% in 2016), reflected by the increase in moderate malnutrition. We also observed an increase in standard obesity among obese patients (from 60.5% to 2012 to 68.2% in 2016). About 80% of our overall population was neither obese nor malnourished.

**Table 1:** Patient characteristics of CHF incident cases between 2012 and 2016

Year	2012	2013	2014	2015	2016
Incident cases	117 552	120 766	123 620	128 069	129 798
Sex (Male)	57,059 (48.5%)	58,427 (48.4%)	60,152 (48.7%)	62,125 (48.5%)	63,269 (48.7%)
Age (SD)	80 +/- 12	80 +/- 12	80 +/- 12	80 +/- 12	80 +/- 12
Malnutrition^a^	9913(8.4%)	11,314 (9.4%)	12,844 (10.4%)	15,057 (11.8%)	15,896 (12.3%)
Severe	4153 (41.9%)	4483 (39.6%)	5005 (39.0%)	5891 (39.1%)	5877 (37.0%)
Moderate	4085 (41.2%)	5036 (44.5%)	6037 (47.0%)	7299 (48.5%)	8029 (50.5%)
Slight	878 (8.9%)	1035 (9.2%)	1118 (8.7%)	1153 (7.8%)	1176 (7.4%)
Unspecified	797 (8.0%)	760 (6.7%)	684 (5.3%)	714 (4.7%)	814 (5.1%)
Obesity^a^	9861 (8.4%)	9868 (8.2%)	10,532 (8.5%)	11,033 (8.6%)	11,546 (8.9%)
Massive	443 (4.5%)	406 (4.1%)	454 (4.3%)	478 (4.3%)	506 (4.4%)
Morbid	1906 (19.3%)	2069 (21.0%)	2238 (21.2%)	2317 (21.0%)	2525 (21.9%)
Standard	5966 (60.5%)	6245 (63.3%)	6928 (65.8%)	7481 (67.8%)	7878 (68.2%)
Unspecified	1546 (15.7%)	1148 (11.6%)	912 (8.7%)	757 (6.9%)	637 (5.5%)

^a^ The figures presented are for all levels included. The detail for each level can be found below

SD: Standard deviation

### Descriptive analysis

**Fig. 1:**
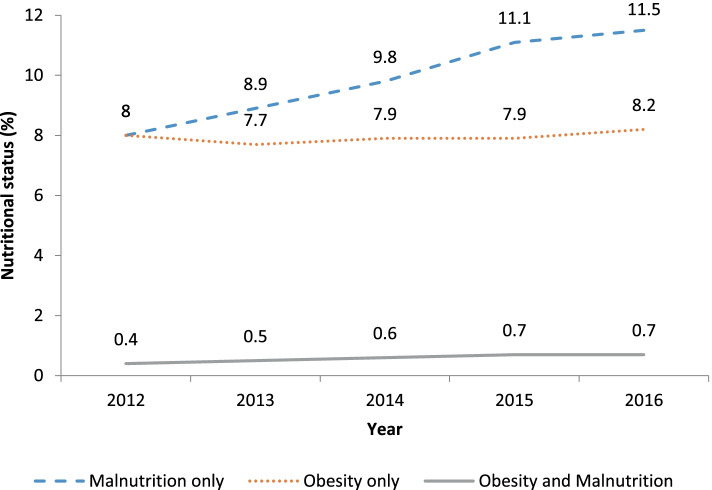
Proportion of the different nutritional statuses among total incident cases of heart failure

As shown in Fig. 1, the proportion of malnutrition among CHF patients increased between 2012 and 2016. There was also a slight increase in the proportion of obesity among the CHF group from 2013 to 2016. The proportion of obesity-malnutrition among CHF patients was very low (less than 1% for all years from 2012 to 2016), but there was a distinct increase from 2012 to 2016.

Among the malnutrition group of CHF patients, severe and moderate malnutrition were the most common statuses (Supplementary Fig. 1), with an increase in the proportion of moderate malnutrition from 2012 to 2016 (Supplementary Fig. 2 A). With regard to obesity, the largest group was standard obesity (BMI 30 to 40 kg/m2), followed by morbid obesity (BMI 40 to 50 kg/m2) (Supplementary Fig. 1 and Supplementary Fig. 2B).

### Bivariate analysis

**Fig. 2:**
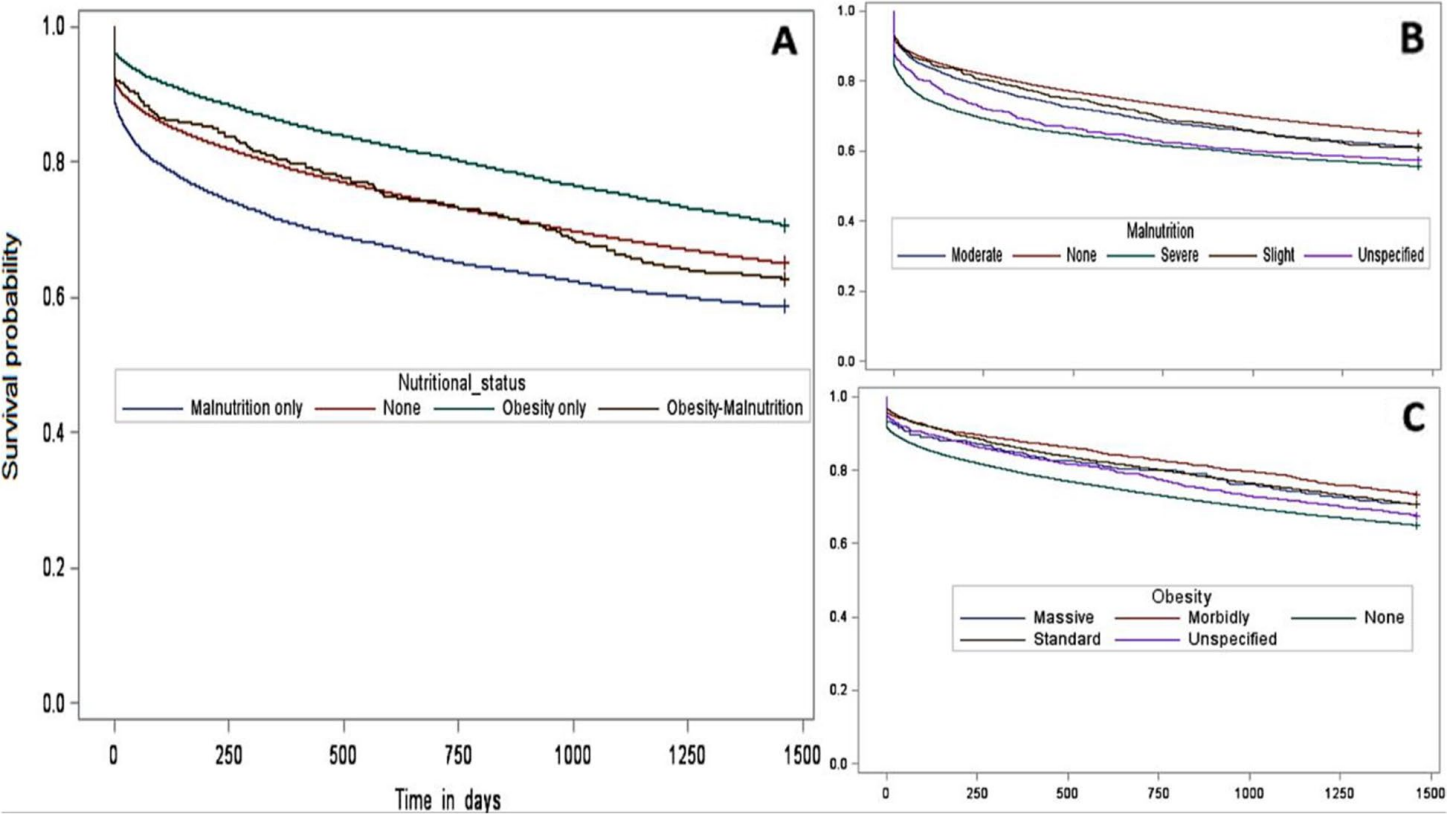
Kaplan-Meier survival curves for the 4-year follow-up cohort, comparing mortality according to the different nutritional status groups (**A**), the different malnutrition level groups (**B**), and the different obesity level groups (**C**)

The observed mortality rate (Table 2) was lowest for the obese group and highest for the malnutrition group (at one year: 13.6% and 28.1%, respectively, and at four years: 29.3% and 41.4%, respectively), compared to the normal status group (at one year: 20.4% and at four years: 35.0%). Although the malnutrition-obesity group appeared to have a higher mortality rate than the normal status group, the difference was not statistically significant (p = 0.82 at one year, p = 0.28 at four years). For survival time, using the Kaplan-Meier method, the malnourished-obese group had a significantly higher probability of survival than the malnourished group and a significantly lower probability than the obese group, while the probability was not significantly different from the non-obese non-malnourished group (Fig. 2 A).

**Table 2:** In-hospital death at one and four years according to nutritional status

	None (a)	Malnutrition only (b)	Obesity only (c)	Malnutrition and obesity (d)	p-value (a) vs. (b)	p-value (a) vs. (c )	p-value (a) vs. (d)
Incident cases from 2012 to 2015	402,197	46,516	38,682	2612			
In-hospital death at one year	82,121 (20.4%)	13,059 (28.1%)	5246 (13.6%)	538 (20.6%)	<0,01	<0,01	0,82
Incident cases in 2012	98,255	9436	9384	477			
In-hospital death at four years	34,350 (35.0%)	3908 (41.4%	2749 (29.3%)	178 (37.3%)	<0,01	<0,01	0,28

In the malnutrition only group, the more severe the malnutrition, the lower the probability of survival (Fig. 2B).

In the obesity only group, all classes of obesity had better survival than non-obese groups (Fig. 2 C).

### Multivariable analysis

After adjustment for age, gender, etiology of CHF, presence of comorbidities such as diabetes, hypertension, dyslipidemia, kidney failure, COPD and other factors (Supplementary Table 2), and taking into account an interaction with time, we found that the obesity only group was at a lower risk of in-hospital death at 4 years (aHR=0.85 [0.81-0.90]) compared to the normal status group (Table 3). On the contrary, the malnutrition only group was at higher risk of in-hospital death (aHR=1.04 [1.004-1.08]). These results were even more marked for the risk of death at 1 year (Table 3).

**Table 3:** Results of multivariable analysis conducted with confounding factor adjustment^a^

	1 year		4 years	
Nutritional status	HR (95%CI)	p-value	HR (95%CI)	p-value
**Global**				
Malnutrition	1.16 (1.14-1.18)	<0.01	1.04 (1.004-1.08)	0.03
Obesity	0.75 (0.73-0.78)	<0.01	0.85 (0.81-0.90)	<0.01
MO	0.85 (0.78-0.93)	<0.01	0.87 (0.74-1.01)	0.07
**Malnutrition level**				
Slight	1.00 (0.93-1.07)	0.94	1.14 (1.02-1.28)	0.02
Moderate	1.11 (1.08-1.15)	<0.01	1.19 (1.12-1.26)	<0.01
Severe	1.45 (1.40-1.49)	<0.01	1.46 (1.38-1.55)	<0.01
Unspecified	1.27 (1.18-1.36)	<0.01	1.22 (1.09-1.36)	<0.01
**Obesity level**				
Standard	0.68 (0.66-0.71)	<0.01	0.74(0.70-0.79)	<0.01
Morbid	0.70 (0.65-0.75)	<0.01	0.65 (0.58-0.72)	<0.01
Massive	0.90 (0.79-1.03)	0.13	0.71 (0.58-0.87)	<0.01
Unspecified	0.91 (0.84-0.98)	<0.01	0.95 (0.86-1.04)	0.25

^a^ Adjusted on age, gender, the etiology of chronic heart failure (Ischemic, dilated or hypertensive cardiomyopathy), the presence of comorbidities such as diabetes, hypertension, dyslipidemia, kidney failure, chronic obstructive pulmonary disease, and the presence of infection and shock during the hospital stay. A complete list of the confounding variables can be found in Supplementary Table 1. MO: Malnutrition and obesity

When we considered the severity of obesity and malnutrition in the obesity only and malnutrition only groups, we found that the more severe the malnutrition, the higher the risk of in-hospital death at 4 years (aHR=1.14 [1.02-1.28] for slight malnutrition and aHR=1.46 [1.38-1.55] for severe malnutrition) compared to people without malnutrition (Table 3). In contrast, we found that all obesity levels decreased the risk of in-hospital death at 4 years (aHR=0.74 [0.70-0.79] for standard obesity, aHR=0.65 [0.58-0.72] for morbid obesity and aHR=0.71 [0.58-0.87] for massive obesity, respectively) compared to people without obesity (cf. Table 2). These results are similar when studying the risk at 1 year (Table 3) although the effect of slight malnutrition and massive obesity were no longer significant (aHR=1.00 [0.93-1.07] and aHR=0.90 [0.79-1.03], respectively).

When excluding ICD-10 codes E660 (Obesity due to excess calories), the survival analysis also showed similar results even if some variables were no longer significant due to the reduction in the statistical power.

## Discussion

Our nationwide study found that patients with a combination of CHF and malnutrition have a higher mortality rate than a control group of patients with CHF and neither malnutrition nor obesity. We observed a relationship between the degree of malnutrition and mortality, in which a higher degree of malnutrition was associated with a higher mortality risk. These findings are consistent with the literature [25, 26].

We also found obesity to be associated with lower mortality for diagnosed CHF patients. This observation has also been described in the literature, and is known as the “obesity paradox” [5, 27]. On closer analysis, we found an inverse relationship between obesity and mortality for BMI ranging between 30 and 50 kg/m². For massive obesity (BMI ≥ 50 kg/m²), mortality was still lower than in the control group, but the protective effect was less pronounced than for patients with a BMI from 40 to 49 kg/m². The inverse association between BMI and mortality in obese heart failure patients found here is discordant with the results of some authors who reported that morbid obesity resulted in higher mortality rates compared to obesity with a BMI of 30 to 40 kg/m^2^ [28]. According to these studies, the lowest mortality rate among obese people is for a BMI of 30 kg/m^2^ to less than 35 kg/m^2,^ or greater than or equal to 30 kg/m^2^ and less than 40 kg/m^2^ [28, 29]. We hypothesize that the difference in mortality for morbid obesity could be explained by differences in diet and physical activity between the French and US population [30]. This hypothesis should be explored by further studies.

CHF patients presenting both obesity and malnutrition, who were the focus of our study, had a lower mortality rate than the malnutrition group but a higher mortality rate than the obesity group. The mortality rate of malnourished-obese patients was not significantly different from the mortality rate of non-obese non-malnourished CHF patients, as if obesity were offset by the effect of malnutrition on mortality. This finding contradicts a previous study showing that sarcopenic obesity increased the risk of mortality compared to sarcopenia alone in patients with cardiovascular disease [14]. There are several potential explanations for this contradiction. The first is that our study focuses on patients with malnutrition obesity and not sarcopenic obesity, which are two similar but nevertheless distinct concepts. The second is that our study only included patients with heart failure and not all cardiovascular diseases. Even so, this result seems to confirm that the paradox of obesity is mainly related to an increase in fat mass rather than to an increase in lean mass, which would be consistent with the Wannamethee et al. [31], who showed that leptin levels are positively associated with a decrease in mortality among heart failure patients. In addition, after adjusting for leptin levels, BMI was no longer a predictor of mortality, therefore suggesting that increased fat mass could have a protective effect as such [31]. Wannamethee’s suggestion that malnourished obese people have a survival rate not significantly different from non-obese non-malnourished people is consistent with our study, indicating that fat mass has a protective effect.

An alternative hypothesis to explain the obesity paradox is a protective effect of the increase in lean mass which is also found in obese people. In obesity, the increased BMI is mostly due to fat mass, but there is also an increase in lean mass, including muscle mass and in particular heart muscle mass, in absolute terms, not percentages. Increased heart muscle mass has already been used as a hypothesis to explain the paradox of obesity during heart failure [32]. This fact is also sustained by recent findings showing that patients with greater levels of cardiorespiratory function had better survival regardless of BMI [33, 34]. The results of our study do not support this hypothesis, since we found that malnourished obese patients have a survival rate that is not significantly different from non-obese non-malnourished patients, as if the fat mass effect compensates for the deleterious effect of lean mass loss, thus indicating the predominant role of fat mass over lean mass in the obesity paradox. Finally, the obesity paradox could be partially explained by an artificially longer follow-up due to early diagnosis of heart failure in obese patients compared to the general population.

### Strengths

Our findings relative to the protective role of obesity and the adverse role of malnutrition are consistent with the literature. Moreover, we studied the effect of the combination of obesity and malnutrition, which have not been previously studied. We found that mortality for this group is not different from the normal group, as if the protective effect of obesity and pejorative effect of malnutrition cancelled each other out. Here, the malnutrition-obesity group was defined as patients with a BMI greater than to 30 and with either low serum albumin or significant weight loss in the last month or 6 months. Seeing as albuminemia reflects intracorporeal lean mass (except in people over 70 years of age [35]), and rapid weight loss mainly affects lean mass [36], malnourished-obese individuals can be considered as individuals with a high fat mass and a low lean mass.

The main strength of our study was the use of a national database for our analysis, with an algorithm that has been validated for CHF identification [37], thus ensuring that all cases of CHF diagnosed in French hospitals were included in the study. The follow-up time was also sufficiently long to detect any significant impact of nutritional status on survival.

### Limits

 In this study, we only measured hospital mortality, even if mortality in France mainly occurs in hospitals. We were only able to assess nutritional status such as malnutrition or obesity via the data available in the PMSI database, and these data are collected for medico-economic purposes. However, we can hypothesize that the exhaustivity is satisfactory because hospitals have a strong financial incentive to collect these data. In particular, in 2009, protein-energy malnutrition was introduced as a French diagnosis-related group classification and included in the hospital tariffs. Thus, slight malnutrition leads to a level 2 stay, whereas moderate or severe malnutrition leads to a level 3 stay with a higher tariff. It is therefore in the interest of establishments to ensure that malnutrition is correctly recorded, and the data is then verified by Medical Information Departments. To avoid misuse, the French Agency for the Management of Hospital Data (ATIH) distributed coding instructions in 2010 so that the Regional Medical Service Departments could take them into account during “external” quality controls. In addition, in order to take into account a potential variation over time, we added a time interaction in our models. Finally, there is no reason for a potential lack of exhaustiveness to occur predominantly in one group in particular (normal, obesity, malnutrition or obesity-malnutrition).

### Perspective

 This study opens new perspectives relative to the obesity paradox. Our results, which suggest the predominant role of fat mass, have yet to be validated. The similar mortality risk in CHF patients with both obesity and malnutrition compared with normal nutritional status should be confirmed and further explored by prospective studies.

## Conclusions

Among CHF patients, malnutrition led to a significant decrease in life expectancy when compared with normal, obese, and obese-malnutrition groups. The greater the nutritional deficiency, the greater the decrease in life expectancy. In contrast, CHF patients with obesity had a significant increase in life expectancy when compared with normal nutritional status, malnutrition, and obese-malnutrition groups. Individuals with all types of obesity had a higher probability of survival than non-obese people. This increase in life expectancy among obese people was highest for a BMI of 40 kg/m2 to 50 kg/m2. Beyond this threshold, life expectancy decreases.

Malnourished-obese heart failure patients were found to have a life expectancy between malnourished patients and obese patients. In addition, they had a life expectancy that was not significantly different from CHF patients with a normal nutritional status.

These results support the hypothesis that the protective effect observed in obese patients may be a result of increasedfat mass. Further studies are needed to explore the possible protective effect of increased body fat during heart failure, and further interventional studies should be conducted to confirm our results. It would also be worthwhile to explore the physiopathological mechanisms that could explain this effect in more detail.

## Supplementary Information

**Additional file 1.**

## Data Availability

The use of these data by our department was approved by the National Committee for data protection. We are not allowed to transmit these data. PMSI data are available for researchers who meet the criteria for access to these French confidential data (this access is submitted to the approval of the National Committee for data protection) from the national agency for the management of hospitalization (ATIH - Agence technique de l’information sur l’hospitalisation). Address: Agence technique de l’information sur l’hospitalisation. 117 boulevard Marius Vivier Merle. 69,329 Lyon Cedex 03.

## Abbreviations

CHF: Chronic heart failure
PMSI: Programme de Médicalisation des Systèmes d’Informations
ICD-10: International Classification of Diseases 10th revision
COPD: Chronic obstructive pulmonary disease
