# Correction: Impact of nutritional status on heart failure mortality: a retrospective cohort study

**DOI:** 10.1186/s12937-022-00811-y

**Published:** 2022-09-29

**Authors:** Nafz Abdoul Carime, Jonathan Cottenet, Guillaume Clerfond, Romain Eschalier, Didier Quilliot, JeanChristophe Eicher, Bertrand Joly, Catherine Quantin

**Affiliations:** 1 Biostatistics and Bioinformatics department (DIM), Dijon University Hospital, Dijon, France;, University of Bourgogne Franche-Comté, Dijon, France; 2 Cardiology Department, CHU Clermont-Ferrand, Clermont-Ferrand, France and Université Clermont Auvergne, CHU Clermont-Ferrand, CNRS, SIGMA Clermont, Institut Pascal, F-63000 Clermont-Ferrand,, France and F-CRIN, INI-CRCT, Nancy, France; 3 Department of Gastroenterology and Inserm NGERE U1256, University Hospital of Nancy, University of Lorraine, 1 Allée du Morvan, 54511 Vandoeuvre-lès-Nancy, France; Nutritional Assistance Department, University Hospital of Nancy, University of Lorraine, Vandoeuvre‑lès‑Nancy, France; 4 Cardiology Department, University Hospital of Dijon, Dijon, France; 5 CHPCB Paray-le-Monial General Hospital, Paray‑le‑Monial, France; 6 Inserm, CIC 1432, Dijon, France; Dijon University Hospital, Clinical Investigation Center, clinical epidemiology/ clinical trials unit, Dijon, France; 7 Biostatistics, Biomathematics, Pharmacoepidemiology and Infectious Diseases (B2PHI), INSERM, UVSQ, Institut Pasteur, Université Paris-Saclay, Paris, France

**Correction: Nutr J 21, 2 (2022)**.

10.1186/s12937-021-00753-x.

Following the publication of the original article [1], the authors reported an error in the Funding section.

The sentence **“This research did not receive any specific grant from funding agencies in the public, commercial, or not-for-profit sectors.”** should be replaced with **“This study was supported by a grant from the French Ministry of Health.”**

The original article has been updated.

## References

[1] Abdoul Carime N, Cottenet J, Clerfond G (2022). Impact of nutritional status on heart failure mortality: a retrospective cohort study. Nutr J.

