# Supplementary material for: Impact of nutritional status on heart failure mortality: a retrospective cohort study
Source: Nutr J. 2022 Jan 6;21:2. doi: 10.1186/s12937-021-00753-x (PMC8734339; doi:10.1186/s12937-021-00753-x)
Supplement: Supplementary file 1 — Additional file 1. [file 12937_2021_753_MOESM1_ESM.docx]

**Supplementary Text 1: Malnutrition coding rules in the French hospital Methodological Guide**

ICD-10 classifies malnutrition states as E40-E46:

E40 Kwashiorkor ; E41 Nutritional marasmus; E42 Kwashiorkor with marasmus; E43 Severe protein-energy malnutrition, unspecified; E44.0 Moderate protein-energy malnutrition ; E44.1 Mild protein-energy malnutrition; E46 Unspecified malnutrition

It uses the generic term malnutrition to refer to a group of conditions resulting from a lack of intake or protein-energy depletion and should therefore be understood in the narrower sense of undernutrition. In a report published in September 2003, the National Agency for Health Accreditation and Evaluation (ANAES) gave a definition: "protein-energy malnutrition results from an imbalance between the body's protein-energy intake and requirements. This imbalance leads to tissue losses with deleterious functional consequences. It is an involuntary tissue loss. "

This definition clearly distinguishes "weight loss [...] from undernutrition by the non-deleterious nature of the weight loss" and its possibly voluntary nature.

In the same report, ANAES established the criteria for assessing protein-energy undernutrition in hospitalised adults of all ages. It considered that the definition provided by the ICD-10, based on statistical criteria, "is not operational in the absence of representative data on weight distribution by age group and sex [...] in France.”

The French High Authority for Health (HAS) revised the criteria for assessing protein-energy undernutrition in the elderly in a report published in April 2007. These criteria are summarised here and recalled by the National Agency for the Management of Hospital Data (ATIH):

1) The diagnosis of undernutrition is based on the presence of at least one of the following criteria.

In patients under 70 years of age:

- weight loss equal to or greater than 10% compared to a value prior to the current hospitalisation, mentioned in a previous medical record;

- weight loss equal to or greater than 5% in 1 month compared to a value prior to the current hospitalisation, as recorded in a previous medical record;

- body mass index (BMI) less than or equal to 17 kg/m²;

- if the test is performed and in the absence of inflammatory syndrome:

- albuminemia less than 30 g/L; (in blood serum, albuminemia (blood albumin level) is about 40-50 g/L.)

- prealbuminemia (transthyretinemia) less than 110 mg/l. Prealbumin has a shorter half-life than albumin (2 days versus 20 days) which makes it more reactive in case of malnutrition (norms 0.1 to 0.4 g/L).

In patients aged 70 years and over:

- weight loss equal to or greater than 5% in 1 month, or equal to or greater than 10% in 6 months;

- BMI less than 21 kg/m2;

- albumin level less than 35 g/l.

2) The diagnosis of severe undernutrition is based on the presence of at least one of the following criteria.

In patients under 70 years of age:

- weight loss equal to or greater than 15% in 6 months, or equal to or greater than 10% in 1 month compared to a value prior to the current hospitalisation, mentioned in a previous medical record ;

- if the test is performed and in the absence of inflammatory syndrome:

- albuminemia less than 20g/l

- prealbuminemia (transthyretinemia) less than 50 mg/l.

In patients aged 70 years and over:

- weight loss equal to or greater than 10% in 1 month, or equal to or greater than 15% in 6 months;

- BMI less than 18 kg/m2;

- albumin level less than 30 g/l.

The use of codes E40 to E46 should be based on these criteria. The clinical evidence for coding one of these codes should be clearly present in the patient's record.

**Supplementary Table 1: ICD-10 codes used for obesity (excluding overweight-specific codes: E6603, E6613, E6683, E6693)**

| **Severity of obesity** | **ICD-10 codes** | **Code wording** |
| --- | --- | --- |
| **Standard** | E6600 | Obesity due to caloric excess in adults with a body mass index [BMI] equal to or greater than 30 kg/m² and less than 40 kg/m², or obesity due to caloric excess in children |
|  | E6610 | Adult drug-induced obesity with a body mass index [BMI] equal to or greater than 30 kg/m² and less than 40 kg/m², or drug-induced obesity in children |
|  | E6620 | Extreme obesity with alveolar hypoventilation, with body mass index equal to or greater than 30 kg/m² and less than 40 kg/m². |
|  | E6680 | Other adult obesity with body mass index [BMI] equal to or greater than 30 kg/m² and less than 40 kg/m², or other childhood obesity |
|  | E6690 | Unspecified adult obesity with body mass index [BMI] equal to or greater than 30 kg/m² and less than 40 kg/m², or unspecified child obesity |
|  | E6604 | Adult caloric overweight with a body mass index [BMI] equal to or greater than 30 kg/m² and less than 35 kg/m², or child caloric overweight |
|  | E6605 | Adult caloric overweight obesity with a body mass index [BMI] equal to or greater than 35 kg/m² and less than 40 kg/m². |
|  | E6614 | Adult drug-induced obesity with a body mass index [BMI] of 30 kg/m² or more and less than 35 kg/m², or child drug-induced obesity |
|  | E6615 | Medicated adult obesity with a body mass index [BMI] equal to or greater than 35 kg/m² and less than 40 kg/m². |
|  | E6624 | Alveolar hypoventilated obesity in adults with a body mass index [BMI] of 30 kg/m² or more and less than 35 kg/m², or alveolar hypoventilated obesity in children |
|  | E6625 | Alveolar hypoventilation obesity in adults with a body mass index [BMI] equal to or greater than 35 kg/m² and less than 40 kg/m². |
|  | E6684 | Other adult obesity with a body mass index [BMI] equal to or greater than 30 kg/m² and less than 35 kg/m², or other childhood obesity |
|  | E6685 | Other adult obesity with a body mass index [BMI] equal to or greater than 35 kg/m² and less than 40 kg/m². |
|  | E6694 | Unspecified adult obesity with a body mass index [BMI] equal to or greater than 30 kg/m² and less than 35 kg/m², or unspecified child obesity |
|  | E6695 | Unspecified adult obesity with a body mass index [BMI] equal to or greater than 35 kg/m² and less than 40 kg/m². |
|  |  |  |
| **Morbidly** | E6601 | Adult caloric overweight obesity with a body mass index [BMI] equal to or greater than 40 kg/m² and less than 50 kg/m². |
|  | E6611 | Medicated adult obesity with a body mass index [BMI] equal to or greater than 40 kg/m² and less than 50 kg/m². |
|  | E6621 | Extreme obesity with alveolar hypoventilation, with body mass index equal to or greater than 40 kg/m² and less than 50 kg/m². |
|  | E6681 | Other adult obesity with a body mass index [BMI] equal to or greater than 40 kg/m² and less than 50 kg/m². |
|  | E6691 | Unspecified adult obesity with a body mass index [BMI] equal to or greater than 40 kg/m² and less than 50 kg/m². |
|  | E6606 | Obesity due to caloric excess in adults with a body mass index [BMI] equal to or greater than 40 kg/m² and less than 50 kg/m². |
|  | E6616 | Medicated obesity in adults with a body mass index [BMI] of 40 kg/m² or more and less than 50 kg/m². |
|  | E6626 | Obesity with alveolar hypoventilation in adults with a body mass index [BMI] equal to or greater than 40 kg/m² and less than 50 kg/m². |
|  | E6686 | Other adult obesity with a body mass index [BMI] equal to or greater than 40 kg/m² and less than 50 kg/m². |
|  | E6696 | Unspecified adult obesity with a body mass index [BMI] equal to or greater than 40 kg/m² and less than 50 kg/m². |
|  |  |  |
| **Massive** | E6602 | Adult caloric overweight obesity with a body mass index [BMI] of 50 kg/m² or more |
|  | E6612 | Drug-induced adult obesity with a body mass index [BMI] of 50 kg/m² or more |
|  | E6622 | Extreme obesity with alveolar hypoventilation, with body mass index equal to or greater than 50 kg/m². |
|  | E6682 | Other adult obesity with a body mass index [BMI] of 50 kg/m² or more |
|  | E6692 | Unspecified adult obesity with a body mass index [BMI] of 50 kg/m² or more |
|  | E6607 | Adult caloric overweight obesity with a body mass index [BMI] of 50 kg/m² or more |
|  | E6617 | Medicated obesity in adults with a body mass index [BMI] of 50 kg/m² or more |
|  | E6627 | Obesity with alveolar hypoventilation in adults with a body mass index [BMI] of 50 kg/m² or more |
|  | E6687 | Other adult obesity with a body mass index [BMI] of 50 kg/m² or more |
|  | E6697 | Unspecified adult obesity with a body mass index [BMI] of 50 kg/m² or more |
|  |  |  |
| **Unspecified** | E6609 | Obesity due to adult caloric excess, body mass index [BMI] not specified |
|  | E6619 | Medicated adult obesity, body mass index [BMI] not specified |
|  | E6629 | Extreme obesity with alveolar hypoventilation in adults, with unspecified body mass index [BMI] |
|  | E6689 | Other adult obesity, body mass index [BMI] not specified |
|  | E6699 | Unspecified adult obesity, body mass index [BMI] not specified |

**Supplementary Table 2: Confounding variables and codes used**

| Diseases | Codes |
| --- | --- |
| *Diseases* | *ICD-10 codes* |
| Left-signs chronic HF | I501 |
| Congestive HF | I500 |
| Cardiogenic shock | R570 |
| Hypertensive cardiomyopathy and HF symptoms | I110 |
| Acute pulmonary oedema | J81 |
| Ischemic cardiopathy | I20 to I25, Z951, Z955, Z8671 |
| Dilated cardiomyopathy | I420 |
| Hypertensive cardiomyopathy and congestive HF | I110, I119, I131, I132, I139 |
| Hypertension | I10 to I13, I15 |
| Diabetes | E10 to E14, N083, O240, H360 |
| Dyslipidemia | E780, E782, E784, E785, E788, E789 |
| Atrial fibrillation | I48 |
| Infection | J14, J15, J18, J22, A46, A40, A41, J168, J170, J110, J128; J129, J172, J440, J690, B377, N300, N308, N309, N390, N110 |
| Anaemia | D50, D62, D63, D510, D511, D513, D518, D519, D520, D528, D529, D531, D538, D539, D464, D461, D462, D591, D594, D599, D641, D642, D648, D649, Y440 |
| Acute coronary syndrome | I200, I210, I211, I213, I214, I219, I220, I231, I238, I248, I249 |
| Acute kidney injury | N170, N171, N178, N179, N990 |
| Other supraventricular arrhythmias | I49 |
| Ventricular tachycardia | I472 |
| Chronic kidney disease | N18, N19, I120, I131 |
| Kidney dialysis | Z49, T824, Y841, Z992 |
| Chronic obstructive pulmonary disease | J40, J41, J42, J44, J432, J438, J439 |
| Pulmonary embolism | I26 |
| Chronic VKA treatment | Z921 |
| Alcohol abuse | K70, F100, F101, F102, F103, F104, F106, F109, G312, G621, I426, K292, K860, T519, X659, Y912, Y919, Z721, F1073 |
| Cachexia | R64 |
| Valvular cardiopathy |  |
| Aortic | I35 |
| Mitral | I34, I05, Q232, Q233 |
| Pulmonary | I37, Q220 à Q223 |
| Several | I08, Q238, Q239 |
| Previous valve replacement | Z952 à Z954 |
| *Procedures* | *CCAM codes* |
| Amine | EQLF003, EQLF001 |
| Coronary angiography | DDQH006, DDQH009 à DDQH015 |
| Cardiac resynchronization therapy | DELF001, DELF014, DELF015, DELF020, DELF902, DELF905 |
| Electrocardiogram | DEQP003, EQRP002 |
| Trans-thoracic echocardiography | DZQM005, DZQM006, DZQJ001, DZQJ008, DZQJ006 |
| Endotracheal intubation | DKMD001 |
| Intensive care unit | DEQP007 |
| Non-invasive ventilation | GLLD019, GLLD012 |

ICD-10: International classification of Diseases 10^th^ revision; HF: heart failure

**Supplementary Figure 1: Flow diagram of the study**

*Patients with an ICD-10 code I50 of heart failure (main or associated diagnosis) in the previous 4 years were excluded.*

861167 Prevalent cases of patients identified with heart failure between 2012 and 2016

619805 Incident cases of patients identified with heart failure between 2012 and 2016

Standard: 32124

Morbid: 10352

Massive: 2148

Unspecified: 4686

Slight: 5023

Moderate: 28694

Severe: 24211

Unspecified: 3566

3530 patients with both malnutrition and obesity

49310 patients with only obesity

61494 patients with only malnutrition

505471 patients with normal status

**Supplementary Figure 2 : proportion of different types of malnutrition (A) and obesity (B) among the malnutrition group and obesity group**
